# Disseminated Cryptococcal Disease in Non-HIV, Nontransplant Patient

**DOI:** 10.1155/2016/1725287

**Published:** 2016-11-09

**Authors:** F. AlMutawa, D. Leto, Z. Chagla

**Affiliations:** ^1^Medical Microbiology Postgraduate Training Program, Pathology and Molecular Medicine, McMaster University, Hamilton, ON, Canada; ^2^Department of Medicine, McMaster University, Hamilton, ON, Canada; ^3^Hamilton Health Sciences, Hamilton, ON, Canada; ^4^Department of Infectious Diseases, McMaster University, Hamilton, ON, Canada; ^5^St. Joseph Healthcare, Hamilton, ON, Canada

## Abstract

Disseminated cryptococcal infection carries a high risk of morbidity and mortality. Typical patients include HIV individuals with advanced immunosuppression or solid organ or hematopoietic transplant recipients. We report a case of disseminated cryptococcal disease in a 72-year-old male who was immunocompromised with chronic lymphocytic leukemia and ongoing chemotherapy. The patient presented with a subacute history of constitutional symptoms and headache after he received five cycles of FCR chemotherapy (fludarabine/cyclophosphamide/rituximab). Diagnosis of disseminated cryptococcal disease was made based on fungemia in peripheral blood cultures with subsequent involvement of the brain, lungs, and eyes. Treatment was started with liposomal amphotericin, flucytosine, and fluconazole as induction. He was discharged after 4 weeks of hospitalization on high dose fluconazole for consolidation for 2 months, followed by maintenance therapy.

## 1. Introduction

Cryptococcus is environmental yeast found worldwide. Two recognized separate species cause the bulk of disease,* Cryptococcus neoformans* and* C. gattii* [1]. There are five different serotypes recognized within these two species. Serotype A is known as* C neoformans* var.* grubii*. Serotype D is* C. neoformans* var.* neoformans*. Serotypes B and C are recognized as the* C. gattii* species [2].

*Cryptococcus neoformans* is found in wild bird and pigeon droppings. It is a significant cause of morbidity and mortality in immunocompromised patients. It mainly infects the HIV positive or transplant recipient population. Other risk factors include the use of high dose steroids, sarcoidosis, and malignancies particularly hematological [3]. Infection is acquired by inhalation of fungal elements from the contaminated soil and is often asymptomatic in normal hosts. Symptomatic disease tends to occur with respiratory symptoms, constitutional symptoms, and a subacute meningitis. We present a case of disseminated cryptococcal disease in a non-HIV, nontransplant patient with good clinical outcomes.

## 2. Case Presentation

**Figure 1:**
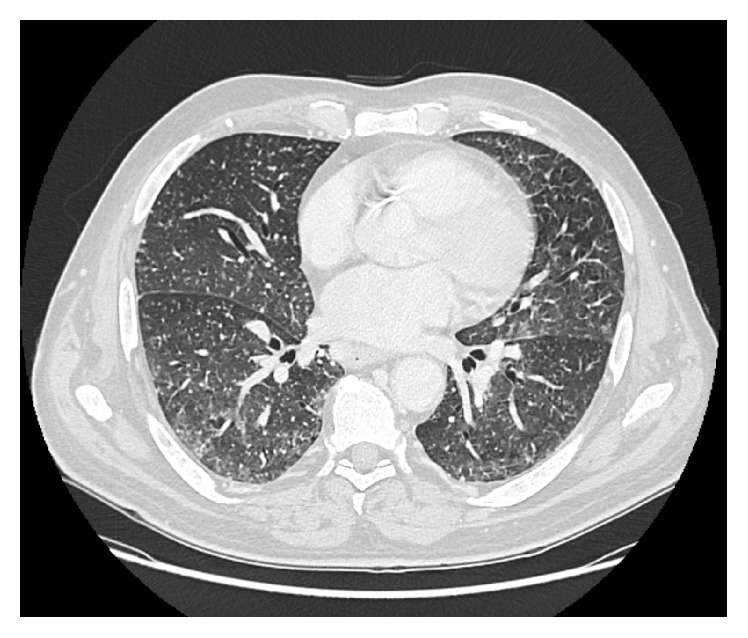
CT chest showing miliary nodules scattered throughout both lungs.

A 72-year-old gentleman presented to hospital for assessment. He had a past medical history significant for chronic lymphocytic leukemia diagnosed 7 years prior to this presentation. He received three cycles of FCR chemotherapy (fludarabine/cyclophosphamide/rituximab), but this was discontinued in the fall of 2013 due to autoimmune hemolytic anemia. He also developed idiopathic thrombocytopenic purpura (ITP) and eventually began a new chemotherapy regimen including cyclophosphamide/vincristine and prednisone. Five cycles into this regimen he developed fevers, night sweats with cough, and mild headaches, without neurologic symptoms. The patient was admitted to the hospital, blood cultures revealed yeast, and caspofungin was started for presumed candidemia. Chest X-ray was normal. When the yeast was identified as* Cryptococcus neoformans*, liposomal amphotericin was started. The identification was based on colonial morphology, capsules seen on India ink, urea test positivity, and MALDI TOF. The initial serum cryptococcal antigen was 1 : 128. His CT chest revealed miliary nodules scattered throughout both lungs with the suggestion of cryptococcal pulmonary infection (Figure 1). CT head showed no focal lesions and symmetric ventricles, with no evidence of hydrocephalus. Lumbar puncture was performed showing normal protein, nucleated cell count 5 with 19 neutrophils and 80 lymphocytes, and positive cryptococcal antigen with a titre 1 : 1, with negative gram stain, bacterial culture, acid fast bacilli stain and TB culture, virology PCR (enterovirus, HSV 1 and 2, VZV), and HIV serology. He had evidence of retinitis bilaterally when assessed by ophthalmology. Flucytosine (100 mg/Kg per day in 4 divided doses) and fluconazole (for eye involvement) were added, in addition to liposomal amphotericin at 6 mg/Kg dose. In hospital he had mild increased ICP (opening pressure between 35–40), with serial therapeutic lumbar punctures. He developed thrombocytopenia approximately 4 weeks into his admission, leading to cessation of amphotericin and flucytosine, with a transition to fluconazole monotherapy. He was discharged after 4 weeks of hospitalization on high dose fluconazole and 800 mg po daily for consolidation for 2 months, followed by 400 mg po daily for maintenance therapy for 1 year. His cryptococcal antigen titers decreased gradually to negative as shown in Table 1. He continues to be on fluconazole 200 mg po daily indefinitely while remaining on prednisone for ITP. A follow-up CT chest done after the 1-year maintenance therapy showed interval resolution of the miliary nodules.

## 3. Discussion

As mentioned above,* C. neoformans* affects mainly immunocompromised individuals worldwide. Serotype A is most commonly isolated, but serotype D is isolated more commonly in European countries [1].* C. gattii* mainly affects immunocompetent people in endemic areas in Australia and Papua New Guinea. Clusters were also reported in British Columbia and in the United States Pacific Northwest. In certain areas such as parts of sub-Saharan Africa, about 10% of HIV cases are infected with* C. gattii* [4]. The infection starts when the fungus is inhaled into the lungs. Infection then spreads to other organs by the circulatory system, mainly to the brain and meninges. Less frequently, other organs such as bones, joints, and skin can be involved. In patients who are not infected with HIV, the disease may occur if they have underlying immunosuppressive conditions, such as Cushing's syndrome, sarcoidosis, hematological malignancies (leukemia or lymphomas), receipt of TNF inhibitors, or organ transplant [2]. Cryptococcal meningitis is a life-threatening infection and requires immediate clinical attention [5].

Cryptococcal polysaccharide antigen can be detected in both serum and CSF. The antigen detection test is very accurate for invasive disease diagnosis [6]. It is nearly 100% sensitive and 96–99.5% specific when serum is tested and 96–100% sensitive and 93.5–99.8% specific on CSF [7]. Amphotericin B is the first line drug for treatment of cryptococcal meningitis. Flucytosine is added to amphotericin B to decrease the rates of treatment failure in severe meningitis, and it is shown to have survival benefit as well [8, 9]. Since fluconazole can penetrate very well into the CSF as indicated in clinical trials, it is used for both the consolidation and suppressive phases of treatment [10, 11]. Fluconazole, however, is not recommended for the induction phase when a polyene can be used as it only has fungistatic activity. It can be used in combination with amphotericin B at 800 mg/day dosing for induction when flucytosine is not available [12].

Non-HIV infected patients are treated with amphotericin B with or without initial flucytosine for 4 weeks for treatment induction. Patients then can receive fluconazole at 400–800 mg/day for 8 weeks for the consolidation phase. The fluconazole dose is then decreased to 200 mg/day for the suppressive phase. Currently there are no well-defined criteria for stopping treatment. Generally, therapy can be stopped when symptoms improve, with a minimum of 2 negative CSF cultures with normal glucose level, or after 6 months to one year of being asymptomatic. Most patients will remain on fluconazole suppressive therapy for at least 6–12 months to prevent relapse, as studies found a 15%–25% relapse rate in the first year after therapy is discontinued [5]. Controlling the patient's underlying disease is the most important factor in prognosis. Non-HIV, nontransplant recipient patients have the worst prognosis, typically due to a delay in diagnosis [13]. It is important to identify patients with high risk for relapse or failure in order to design a specific or prolonged antifungal regimen for their treatment course.

A literature review of the reported cases on disseminated* cryptococcal neoformans* infection in HIV negative patients between 2010 and 2015 is presented in Table 2. Most patients are males with underlying immunosuppression such as transplant and hematological malignancy. Patients received different modalities of treatment in this review as shown in the table. Outcome varies between resolution, relapse, and death.

In summary, we present a non-HIV, nontransplant, immunosuppressed individual with CLL and chemotherapy with disseminated cryptococcal disease involving eyes, brain, and lungs, with positive blood cultures. He was treated with amphotericin, flucytosine, and fluconazole, with a transition to fluconazole consolidation and maintenance therapy, which the patient currently remains on. Disseminated cryptococcal infection is a potential cause of serious morbidity and mortality. It can affect both immunocompromised and immunocompetent people. Delayed diagnosis in unusual hosts, such as the non-HIV, nontransplant population, may lead to unfavourable outcomes.

## Figures and Tables

**Table 1:** Patient cryptococcal titre trend during follow-up period.

Serum cryptococcal titre		Spinal fluid cryptococcal titre	
May 2014	1 : 128	May 2014	1 : 1
June 2014	1 : 64	June 2014	Negative
July 2014	1 : 64	July 2014	Negative
August 2014	1 : 32		
November 2014	1 : 16		
February 2015	1 : 4		
April 2015	Negative		

**Table 2:** Review of HIV negative disseminated cryptococcal case reports.

Age	Sex	Underlying condition	Presentation	Microbiology	Treatment	Outcome	References
62	Male	Renal transplant on cyclosporine, azathioprine, and prednisone	Miliary pulmonary cryptococcus	Positive blood cultures	Fluconazole	Did well and stable	[14]
43	Male	Renal transplant on cyclosporine, mycophenolate mofetil, and prednisone	Cellulitis	Positive blood cultures	Amphotericin lipid complex at 6 mg/Kg for 2 weeks and then fluconazole for 3 months	No recurrence at 4-month follow-up	[15]
34	Male	Nephrotic syndrome on prednisone	Bilateral LL ulcers	Blood, sputum, and biopsy cultures	Fluconazole	Death	[16]
26	Female	No significant past history	Crohn's disease	Colon and sputum	Fluconazole for 5 weeks	Improved	[17]
7	Male	No significant past history	Hepatosplenomegaly, LAP, bilateral choroiditis, and skin lesions	Blood, CSF, and skin biopsy cultures	Amphotericin and fluconazole	Remission	[18]
79	Male	COPD and CAD	Splenomegaly, LAP, and respiratory symptoms	Sputum cultures and bone marrow PCR	Amphotericin and fluconazole	Death	[19]
28	Male	No significant past history	Hepatosplenomegaly, LAP, and lymphopenia	Lymph node biopsy culture	Amphotericin and fluconazole	Death	[20]
70	Male	No significant past history	Thigh nodule, chest, and brain lesions	Biopsy of thigh nodule and sputum cultures	Amphotericin and fluconazole	Resolution of his symptoms and lesions at 1-year follow-up	[21]
65	Female	NHL on CHOP- rituximab	Meningitis and respiratory symptoms	Blood, pleural fluid, and CSF cultures	Amphotericin, flucytosine, and fluconazole	Remission	[22]
63	Male	Diabetes mellitus, AHA-steroid, and splenectomy	Headache, photophobia, decreased vision, panuveitis, and lung/brain lesions	Lung biopsy culture	Amphotericin, flucytosine, and fluconazole for 1 year	Improved	[23]

LAP, lymphadenopathy; NHL, non-Hodgkin's lymphoma; CHOP, C: cyclophosphamide, H: doxorubicin hydrochloride (adriamycin), and O: vincristine (Oncovin); PCR, polymerase chain reaction; mg, milligrams; Kg, kilograms.
